# Formation, Structural Characteristics and Functional Properties of Quercetin–Oat β-Glucan Complex

**DOI:** 10.3390/foods15101825

**Published:** 2026-05-21

**Authors:** Wenjing Xie, Wenjun Wang, Xinlu Feng, Raojun Zheng, Lingli Chen, Ningmeng Ding, Qiujun Chen, Suyun Lin

**Affiliations:** 1 Jiangxi Key Laboratory of Natural Products and Functional Food, College of Food Science and Engineering, Jiangxi Agricultural University, Nanchang 330045, China; 18296204542@163.com (W.X.); wwjun9999@jxau.edu.cn (W.W.); fxl18864777292@163.com (X.F.); chenlingli89@163.com (L.C.); 13686090825@163.com (N.D.); 18522776979@163.com (Q.C.); 2 School of Chemistry and Environmental Science, Shangrao Normal College, Shangrao 334001, China; raojunzheng@126.com

**Keywords:** quercetin, oat β-glucan, Complex interaction, α-amylase inhibition, viscoelasticity

## Abstract

Quercetin (QE), a flavonol-type polyphenol, and oat β-glucan (OβG), a soluble dietary fiber, are natural active ingredients with the potential to reduce the risk of diabetes. OβG slows starch digestion by modifying chyme viscosity, while QE inhibits digestive enzyme activity. This study aimed to explore the formation mechanism and structural characteristics of QE-OβG complexes, as well as their functional properties in terms of viscosity and amylase inhibitory activities. It was found that QE and OβG formed stable non-covalent complexes via hydrogen bonding and hydrophobic interactions. At a mass ratio of 0.6, the binding capacity was relatively high with a moderate aggregation degree, representing a balanced interaction state. Changes in turbidity and particle size indicated that different environmental factors (pH, temperature, ionic strength) exert differential effects on the aggregation behavior of the complex. In addition, the complex exhibited a unique fibrous-block morphology, enhanced thermal stability, improved starch system viscoelasticity, and stronger mixed-type reversible α-amylase inhibition (IC_50_ = 2.629 mg/mL). This study clarifies the interaction mechanism between QE and OβG, provides a reliable theoretical basis for the development of novel hypoglycemic foods, and offers new insights into multi-component regulation strategies for slow-digestion food design.

## 1. Introduction

Quercetin (QE) is a typical flavonol-type polyphenol widely found in fruits and vegetables [1]. Studies have clearly confirmed that QE not only possesses excellent properties such as antihypertensive [2] and anti-diabetic [3] physiological activities, but also has the ability to inhibit the activity of starch digestive enzymes, which is beneficial for slowing down starch digestion [4]. It is thus an active ingredient with great potential for application in the development of functional foods.

Oat β-glucan (OβG) is a soluble dietary fiber found in oats which belongs to the class of viscous polysaccharides. OβG has prominent physiological functions, including lowering auxiliary blood glucose [5] and relieving constipation [6]. Additionally, it can slow down starch digestion by increasing chyme viscosity [7].

Whether added directly or exposed to polyphenolic compounds from other foods during food processing, storage, and digestion, the coexistence of polysaccharides and polyphenols is a common phenomenon in daily diets. Studies have indicated that non-covalent interactions can occur between polysaccharides and polyphenols, and these interactions may affect their functional activities, thereby influencing the nutritional efficacy of food [8]. For instance, Li et al. reported that pectin and anthocyanins in wine can form noncovalent complexes, primarily through hydrogen bonding and electrostatic interactions. These interactions were found to modulate the astringency, color stability, and aroma release of wine during their investigation of grape polysaccharides and wine sensory-active compounds, including polyphenols and volatile components [9]. Moreover, the interactions between polyphenols and polysaccharides are also affected by external environmental factors, including pH, temperature, ion type and concentration [10]. Tan et al. [11] found that high hydrostatic pressure treatment exposed pectin’s charged groups, strengthening the electrostatic interactions and binding affinity between cyanidin-3-glucoside and blueberry pectin. Quercetin (QE) possesses multiple hydroxyl groups and aromatic rings in its molecular structure, which enable it to interact readily with polysaccharides such as β-glucan that contain abundant hydroxyl groups on their backbone chains. Such intermolecular interactions are speculated to facilitate the formation of stable complexes, thereby altering the structural and functional properties of the system. Furthermore, QE exhibits notable inhibitory activity against α-amylase, while oat β-glucan (OβG) exerts glycemic regulatory effects mainly through its high viscosity. Accordingly, the combined use of QE and OβG is expected to provide complementary functional advantages, showing considerable potential for combining enzyme inhibition with viscosity-mediated regulation of starch digestion. The investigation of complexes is a highly suitable route for the development of dietary supplements and glycemic-control-related pharmaceutical products. Nevertheless, the interaction mechanism between QE and OβG, and the comprehensive effects of their complexation on starch-related functional properties, remain to be investigated.

Therefore, this study explores the formation mechanism and structural characteristics of the complex of QE and OβG, as well as its functional properties in terms of viscosity and amylase inhibitory activities, both of which are highly related to its ability to slow down starch digestion. This study is expected to be of great significance for investigating the functional changes of components in complex food systems and guiding people to rationally combine dietary sources. Furthermore, it provides a theoretical basis for enhancing the industrial value and health benefits of dietary fiber and polyphenols.

## 2. Materials and Methods

### 2.1. Materials

OβG (95%, (C_6_H_10_O_5_)n, 20,000~40,000 Da, derived from oats) was purchased from Zhongkang Food Co., Ltd. (Guangzhou, China). QE (98%, C15H10O7, 302.24) and porcine pancreatic α-amylase were obtained from Macklin Biochemical Technology Co., Ltd. (Shanghai, China). Acarbose was purchased from Yuanye Bio-Technology Co., Ltd. (Shanghai, China). 3,5-Dinitrosalicylic acid (DNS) was purchased from Solarbio Science & Technology Co., Ltd. (Beijing, China). Corn starch was purchased from Haitian Flavouring and Food Co., Ltd. (Foshan, China). Sodium hydroxide, hydrochloric acid, potassium bromide, and sodium chloride were of analytical grade. Ultrapure water was used throughout the experiments.

### 2.2. Preparation of QE-OβG Complex

The preparation process of the QE-OβG complex is shown in Figure S1. OβG stock solution (200 µg/mL) was prepared by dispersing 0.040 g of OβG powder in 200 mL of ultrapure water, followed by magnetic stirring at 60 °C for 2 h in a water bath (SHJ-A6, Changzhou Yining Laboratory Instrument Factory, Changzhou, China) to ensure complete hydration. After cooling to ambient temperature, the solution was centrifuged at 8000× *g* for 15 min using an ultracentrifuge (OPTIMA MAX-XP, Beckman Coulter, Pasadena, CA, USA) to remove insoluble residues, and the supernatant was collected as the OβG stock solution. The QE stock solution (200 µg/mL) was prepared by dissolving 0.010 g of QE powder in 50 mL of ultrapure water with continuous magnetic stirring at ambient temperature for 2 h to ensure complete dissolution. The solubility of QE at this concentration was sufficient to form a true solution without the need for co-solvents. The final pH of the QE stock solution was approximately 6.5, which was determined using a pH meter. After preparation, the QE solution was kept in the dark to avoid degradation by light.

The QE stock solution was then diluted to final concentrations of 20, 40, 80, 120, 160 and 200 µg/mL. Each diluted QE solution was then mixed with the OβG stock solution at varying mass ratios (0.1, 0.2, 0.4, 0.6, 0.8, and 1.0) and incubated at ambient temperature for 0.5 h with continuous stirring. The mixture was maintained in the dark during incubation to preserve the stability of QE.

A single-factor experimental design was adopted. The investigated factors included QE/OβG mass ratio (0.1, 0.2, 0.4, 0.6, 0.8, 1.0), pH (2, 3, 4, 5, 6, 7), temperature (4 °C, 25 °C, 40 °C, 55 °C, 70 °C, 85 °C), and ionic strength (0 M, 0.1 M, 0.2 M, 0.3 M, 0.4 M, 0.5 M). These levels were chosen based on preliminary experiments, as well as typical food processing and simulated digestive conditions reported in the literature.

### 2.3. Turbidity Measurement

The turbidity of the QE-OβG complex was determined by measuring the absorbance at 600 nm using a UV-Visible spectrophotometer (U-T1810, Shanghai Yipu Instrument Manufacturing Co., Ltd., Shanghai, China), with ultrapure water serving as the blank reference. Absorbance values at 600 nm were used directly as turbidity indicators.

### 2.4. Particle Size and Zeta Potential Measurement

The particle size distribution (including average particle diameter and polydispersity index, PDI) and surface charge (ζ-potential) of QE-OβG complexes at different QE/OβG mass ratios were analyzed using a dynamic light scattering potentiometric particle size analyzer (Malvern Instruments Ltd., Malvern, UK).

### 2.5. Flavonoid Binding Capacity Assay

QE and OβG were mixed at specified mass ratios and incubated at 25 °C with continuous stirring at 300 rpm for 2 h to ensure complete interaction between the components. After incubation, the resulting complex suspension was then centrifuged using an ultrafiltration tube at 8000× *g* for 15 min. The absorbance of the filtrate was measured at the characteristic absorption peak of QE. The QE concentration in the filtrate was determined using a pre-established standard calibration curve: Y = 0.001X + 2.7635 (R^2^ = 0.9905), where the QE concentration ranged from 10 to 200 µg/mL. The amount of QE bound to OβG was calculated using the following formula [12]:

Binding Capacity (µg/mg) = (M_QE_ − CV)/M_OβG_ (1)

where M_QE_ means the total initial mass of QE initially (ug), C means the concentration of free QE in the filtrate (µg/mL), V means the total volume of filtrate (mL), and M_OβG_ is the total mass of OβG (mg).

### 2.6. Effects of Environmental Factors on QE-OβG Complex

Based on the preliminary results, the QE-OβG complexes prepared at a mass ratio of 0.6 were selected to investigate the effects of environmental factors (pH, temperature, and ionic strength) on their properties. This ratio was chosen because it represents the point at which a significant increase in turbidity, binding capacity, and particle size was observed, suggesting a notable change in complex formation.

#### 2.6.1. Effect of pH

The pH of the QE-OβG complex solutions was adjusted to 2.0, 3.0, 4.0, 5.0, 6.0, and 7.0 with 0.5 M using 0.5 M HCl or 0.5 M NaOH, monitored with a pH meter (pHS-3C, OUSTOR Industrial Co., Ltd., Shanghai, China). After adjustment, the samples were incubated at ambient temperature for 30 min before subsequent analyses.

#### 2.6.2. Effect of Temperature

The QE-OβG complex solutions were incubated in thermostated environments at 4 °C, 25 °C, 40 °C, 55 °C, 70 °C, and 85 °C for 30 min. Samples treated above 25 °C were cooled to ambient temperature prior to measurement.

#### 2.6.3. Effect of Ionic Strength

Sodium chloride (NaCl) was added to the QE-OβG complex solutions to achieve final NaCl concentrations of 0 M, 0.1 M, 0.2 M, 0.3 M, 0.4 M, and 0.5 M. The samples were stirred to ensure complete dissolution and then incubated at ambient temperature for 30 min before analysis.

### 2.7. Structural Characterization of QE-OβG Complexes

QE-OβG complexes prepared at a QE/OβG mass ratio of 0.6 were centrifuged to collect the precipitate using an ultracentrifuge, which was then washed three times with ultrapure water and centrifuged again to remove free QE. The washed precipitate was freeze-dried using a Vacuum Freeze Dryer (NFD-1C-80, Tianjin ZSBIO Co., Ltd., Tianjin, China) to obtain QE-OβG complex powder for structural characterization.

#### 2.7.1. Cryo-Scanning Electron Microscopy (Cryo-Sem)

The morphology of the freeze-dried QE-OβG complex powder was observed using a cold field emission scanning electron microscope (JSM-7500F, JEOL Ltd., Tokyo, Japan), following a modified method from Xiao et al. [13]. Briefly, the powder was dispersed on a conductive adhesive-coated sample holder. The holder was rapidly frozen in liquid nitrogen slush for 30 s, transferred under vacuum to a preparation chamber, and warmed to −100 °C on a cryo-stage to sublime surface frost for 3 min. The sample was then sputter-coated with gold (10 mA, 48 s) before imaging.

#### 2.7.2. Fourier Transform Infrared Spectroscopy (FTIR)

Following the methods adapted by Zheng et al. [14], the complex powder was ground and passed through a 200-mesh sieve. It was then mixed with KBr at a 1:100 (*w*/*w*) ratio, thoroughly ground, and pressed into a pellet. Spectra were acquired using a Fourier transform infrared spectrometer (Nicolet IS10, Thermo Fisher Scientific Inc., Waltham, MA, USA) with 32 scans over a range of 4000–400 cm^−1^ at a resolution of 4 cm^−1^, using a blank KBr pellet for background correction.

#### 2.7.3. Differential Scanning Calorimetry (DSC)

DSC analysis was conducted using a differential scanning calorimeter (DSC200F3, Netzsch GmbH, Selb, Germany), modified from Peng et al. [15]. Approximately 5 mg of freeze-dried QE-OβG complex powder was sealed in an aluminum crucible, with an empty crucible as reference. The sample was heated from 25 to 200 °C at a rate of 10 °C/min under a nitrogen atmosphere (purge gas flow: 20 mL/min; protective gas flow: 60 mL/min).

### 2.8. Rheological Properties of QE-OβG Complexes

Specified amounts of QE and OβG were added to corn starch, keeping the total dry powder mass at 3 g. Then, 25 mL of ultrapure water was added and the mixture was stirred thoroughly to achieve a uniform suspension. Three sample series were prepared: one with OβG mass fractions of 1%, 2%, and 3% relative to the total powder mass; one with QE mass fractions of 0.4%, 1.2%, and 2.4% relative to the total powder mass; and another with QE/OβG mass ratios of 0.4, 0.6, and 0.8. A pure corn starch suspension served as the blank control. All suspensions were gelatinized in a 95 °C water bath for 30 min to obtain starch pastes. Following a modified method from Li et al. [16], the hot paste was immediately transferred to a discovery hybrid rheometer (ARES-G3, Waters Co., Ltd., Milford, MA, USA), Measurements were conducted using a parallel plate geometry (diameter: 40 mm; gap: 1000 µm) at 25 °C.

#### 2.8.1. Static Rheological Measurement

The static shear flow behavior was characterized by measuring apparent viscosity (η) and shear stress (τ) across shear rates (γ) from 0 to 100 s^−1^ and back to 0 s^−1^, with an optimal stress of 0.028 Pa. Data were fitted to the power-law model:

τ = K × γ^n^ (2)

Here, τ represents shear stress (Pa), K represents the consistency coefficient (Pa·s^n^), γ represents the shear rate (s^−1^), and n represents the flow behavior index.

#### 2.8.2. Dynamic Rheological Measurement

The dynamic viscoelasticity of the material was characterized by measuring the evolution of storage modulus (G′), loss modulus (G″), and phase angle (δ) as a function of frequency (ranging from 0.1 to 50 Hz). During the frequency sweep, the optimal stress for linear viscoelastic response was first determined to be 0.028 Pa using the TRIOS 4.2.1 software, ensuring the system remained within the linear viscoelastic region. Additionally, during the rheological measurements, dimethyl silicone oil was applied to the edge of the starch paste to prevent moisture loss and eliminate its potential impact on the test results.

### 2.9. Starch Digestive Enzyme Inhibition Properties of QE-OβG Complexes

#### 2.9.1. α-Amylase Activity Inhibition Assay

The α-amylase inhibitory activity was determined using the 3,5-dinitrosalicylic acid (DNS) colorimetric method.

Corn starch (10 mg/mL) served as the substrate. Porcine pancreatic α-amylase (PA) was dissolved in ultrapure water (0.5 mg/mL). Reaction mixtures contained 0.5 mL of α-amylase solution, 0.5 mL of inhibitor solution (QE, OβG, or QE-OβG complex at 0.5, 1, 2, 4, or 8 mg/mL), and 0.5 mL of phosphate buffer (PBS, pH 7.2). After pre-incubation at 37 °C for 10 min, 0.5 mL of starch solution was added and the reaction proceeded at 37 °C for another 10 min. The reaction was terminated by adding 0.5 mL of DNS reagent, followed by heating in a boiling water bath for 10 min and cooling in an ice-water bath. The absorbance was measured at 540 nm using a microplate reader (SpectraMax M2, Shanghai Molecular Devices Co., Ltd., Shanghai, China). Acarbose was used as a positive control. All experiments were performed in triplicate [17]. The inhibition rate was calculated using the following formula:

C = (1 − (A_1_ − A_2_)/(A_3_ − A_4_)) × 100% (3)

where C represents the α-amylase inhibition rate (%), A_1_ represents the absorbance of the test group, A_2_ represents the absorbance of the test blank group, A_3_ represents the absorbance of the control group, A_4_ represents the absorbance of the background group.

#### 2.9.2. Analysis of α-Amylase Inhibition Type

The half-maximal inhibitory concentration (IC_50_) was determined by plotting inhibition rates against inhibitor concentrations.

To assess inhibition reversibility, reactions were conducted with fixed substrate (10 mg/mL) and two inhibitor concentrations (4 and 8 mg/mL, expressed on a total-mass basis), each tested against a gradient of α-amylase concentrations (0.1–0.8 mg/mL). The initial reaction rate (v, change in absorbance per minute) was plotted against enzyme concentration.

To determine the inhibition type (competitive, non-competitive, or mixed), the enzyme concentration was fixed at 0.5 mg/mL, and two inhibitor concentrations (1 and 4 mg/mL) were tested against a range of substrate concentrations (5, 7.5, 10, 12.5, and 15 mg/mL). Lineweaver–Burk double reciprocal plots were drawn with the reciprocal of the enzyme reaction rate (1/ν) as the ordinate and the reciprocal of the substrate concentration (1/[S]) as the abscissa, and changes in the maximum reaction rate (V_max_) and Michaelis constant (K_m_) were analyzed.

### 2.10. Data Analysis

All experiments were performed in at least triplicate (*n* = 3). Prior to one-way analysis of variance (ANOVA), the normality of data distribution was verified by the Shapiro–Wilk test, and the homogeneity of variances was verified by Levene’s test. All data conformed to the assumptions of normality and homogeneous variance. Significant differences among groups were determined by ANOVA followed by Duncan’s multiple range test at a significance level of *p* < 0.05. Graphs were generated using the Origin 2021 software.

## 3. Results and Discussion

### 3.1. Effect of the Mass Ratio of QE to OβG on the Formation of QE-OβG Complexes

Given that neither OβG nor QE exhibits significant absorption at 600 nm, this wavelength was selected to measure turbidity via absorbance without spectral interference. Turbidity can thus serve as an indicator of complex formation through light scattering [18]. However, turbidity alone is not a specific indicator of complex formation, as it may also arise from nonspecific aggregation or precipitation. Therefore, turbidity results were interpreted in combination with particle size and binding capacity measurements to provide more reliable evidence for complex formation.

**Figure 1:**
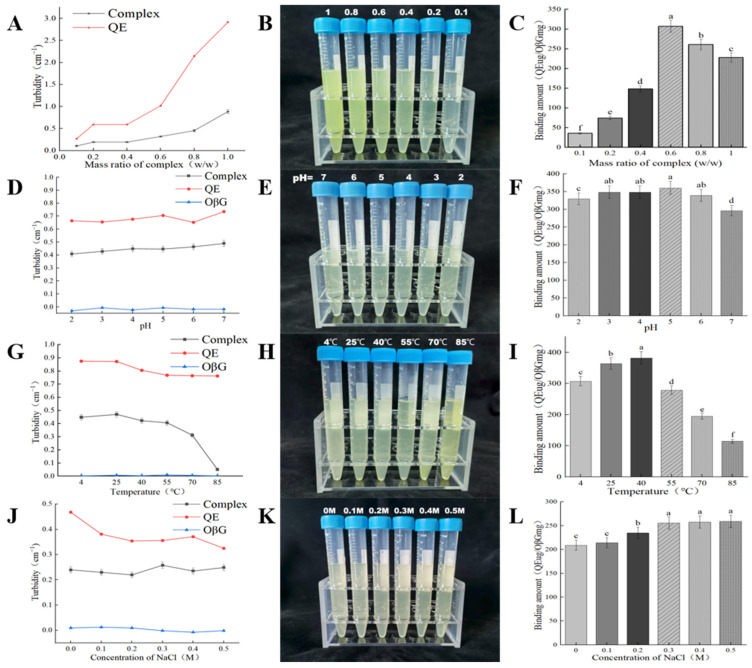
Effects of mass ratio (0.1, 0.2, 0.4, 0.6, 0.8, and 1.0), pH (2.0, 3.0, 4.0, 5.0, 6.0, and 7.0), temperature (4 °C, 25 °C, 40 °C, 55 °C, 70 °C, and 85 °C), and ionic strength (NaCl concentration: 0 M, 0.1 M, 0.2 M, 0.3 M, 0.4 M, and 0.5 M) on QE-OβG complex formation. Note: (**A**,**D**,**G**,**J**): Turbidity; (**B**,**E**,**H**,**K**): Visual appearance of the complex solution; (**C**,**F**,**I**,**L**): QE binding capacity to OβG (μg/mg). Notes: Different letters in a single column denote significant differences at *p* < 0.05.

The visual appearance and turbidity of mixtures at different mass ratios are presented in Figure 1A,B. With the OβG concentration fixed at 200 µg/mL, increasing the QE concentration led to a gradual rise in turbidity. A marked increase in both color intensity and turbidity was observed at a mass ratio of 0.6, suggesting the onset of significant aggregation and light-scattering complex formation. The amount of QE bound to OβG followed a similar trend (Figure 1C). Binding increased slightly as the mass ratio rose from 0.1 to 0.4, then sharply between ratios of 0.4 and 0.6. At higher ratios (0.8 and 1.0), the binding amount plateaued or slightly decreased. This phenomenon may be attributed to the fact that the binding sites on OβG were nearly saturated at this time, while the non-covalent interaction between polyphenols and polysaccharides was reversible and the binding stability was relatively weak, which led to dissociation of part of the unstably bound free QE from the binding sites of β-glucan or the dynamic change of the secondary binding state [19]. These results confirm that QE and OβG can form complexes and that the extent of complexation is influenced by the QE concentration.

Particle sizes and zeta potentials for the complexes are shown in Table 1. As the QE concentration increases, the particle size of the formed complexes also increases, indicating enhanced aggregation and interaction between QE and OβG at higher concentrations. This may be attributed to the increased availability of QE molecules, which facilitates more extensive association with OβG chains and promotes the formation of larger aggregates. Zeta potential values for all complexes were relatively low, ranging from −11 to −18 mV, and did not show a consistent trend with mass ratio. This suggests that electrostatic interactions are not the primary driving force for complex formation. This finding aligns with studies indicating that complexes between neutral polysaccharides and polyphenols are predominantly stabilized by hydrogen bonding and hydrophobic interactions, rather than electrostatic forces [20].

### 3.2. Effect of Environmental Factors on QE-OβG Complexes

Based on the experimental results of the complex mass ratio, both turbidity binding capacity and particle size showed a significant increase trend at a mass ratio of 0.6. This suggests that aggregation occurs between the complexes at a mass ratio of 0.6, forming a more stable complex. However, these parameters primarily reflect the extent of aggregation rather than the structural quality or uniformity of the complexes. At higher QE levels, excessive QE may lead to partial self-association or oversaturation of available binding sites on OβG, resulting in less controlled and potentially heterogeneous structures. Therefore, the intermediate mass ratio of 0.6 was selected not simply as the point of maximum aggregation, but as a relatively balanced interaction state where sufficient binding occurs without excessive aggregation. This condition is more likely to yield structurally stable and functionally relevant complexes. Therefore, the QE-OβG complex with a mass ratio of 0.6 was selected for subsequent experiments to investigate the effects of environmental factors on QE-OβG complex interactions. These environmental factors included pH, temperature, and ionic strength.

#### 3.2.1. Effect of pH on QE-OβG Complex Formation

The visual appearance, turbidity, and QE binding capacity of the complex at pH levels from 2.0 to 7.0 are shown in Figure 1D–F. Turbidity gradually decreased as pH was lowered from 7 to 2 (Figure 1D,E). The QE binding capacity increased from pH 2 to 5, then decreased at pH 6 and 7 (Figure 1F). This decrease may result from QE undergoing accelerated degradation and instability under neutral to alkaline pH conditions [21], weakening the interaction forces with OβG and consequently reducing the binding amount. Furthermore, QE is incompletely ionized at lower pH conditions, resulting in a relatively minor contribution of electrostatic interactions to its adsorption. This aligns with the viewpoint proposed by Liu et al. [22], suggesting that at low pH, van der Waals forces and hydrogen bonding contribute more significantly to binding than electrostatic interactions. Since pH primarily influences changes in the surface potential of dextran and the ionization of QE—thereby determining the magnitude of electrostatic interactions—the adsorption of QE is not significantly affected by pH.

The effect of pH on the particle size of QE-OβG complexes is presented in Table 1, where the average particle size of the complex increases with decreasing pH. The pH-dependent formation and aggregation behavior of the complex can be attributed to changes in the ionization state of the hydroxyl group in the polyphenol molecule: at low pH, the polyphenol hydroxyl group becomes protonated, significantly enhancing hydrogen bonding interactions between QE and OβG, thereby promoting the formation of larger aggregates. Conversely, as pH increases, QE molecules gradually dissociate, leaving only partially dissociated hydroxyl groups capable of interacting with OβG. Simultaneously, the dissociated polyphenol molecules carry negative charges in solution, generating electrostatic repulsion that further disrupts their interactions. Consequently, higher pH conditions inhibit complex formation.

#### 3.2.2. Effect of Temperature on QE-OβG Complex Formation

The effect of temperature on QE-OβG complex formation is illustrated in Figure 1G,H, which displays the turbidity and appearance of QE, OβG, and their complex across temperatures ranging from 4 °C to 85 °C. The turbidity curve (Figure 1G) reveals a clear trend: QE turbidity slightly decreases with rising temperature (but remains relatively stable overall); OβG turbidity stays nearly constant; and complex turbidity gradually decreases as temperature decreases. This may be attributed to the non-covalent bonds between phenolic compounds and polysaccharides, primarily formed through hydrogen bonds and hydrophobic interactions. The hydroxyl group is the main functional group involved in hydrogen bonding between QE and OβG, and it is significantly affected by temperature [23]. Consequently, as temperature increases, the hydroxyl bonds between QE and OβG weaken, making it difficult to maintain the original aggregated state. This alteration in the dispersion of the system leads to a decrease in turbidity. The highest QE binding was seen at 40 °C in Figure 1I, and binding decreased significantly with increasing temperature, which may result from temperature-dependent QE stability, as high-temperature heat treatment degrades QE [24]. Consequently, the QE binding capacity significantly decreases at higher temperatures, consistent with the findings of Liu et al. [25].

The effect of temperature on the particle size of QE-OβG complexes is summarized in Table 1. The average particle size of the complex first increases, then decreases with rising temperature. This indicates that moderate temperatures may promote molecular interactions and aggregation, while excessive heating could disrupt the formed structures or lead to partial disassembly. Meanwhile, the zeta potential values fluctuated irregularly with temperature, indicating that electrostatic interactions are not the dominant driving force in QE-OβG complex formation. These results further support that the interactions between QE and OβG are primarily governed by non-covalent forces such as hydrogen bonding and hydrophobic interactions.

#### 3.2.3. Effect of Ionic Strength on QE-OβG Complex Formation

The effect of ionic strength on the turbidity and appearance of the QE-OβG complexes is shown in Figure 1J,K. The complex solution remained transparent throughout the ionic strength range (0–0.5 M), and its turbidity was not affected by the change in ionic strength to produce a significant difference. Secondly, the effect of NaCl concentration (ionic strength) on the binding of QE to OβG was determined. Referring to related research reports on the binding of polysaccharides and polyphenols, it can be seen that if the amount of polysaccharides bound to polyphenols rises with the ionic strength, it usually implies that electrostatic and hydrophobic interactions play an important role in the binding process of the two [26]. As Figure 1L shows, QE-OβG binding was low at 0 and 0.1 M NaCl, rose sharply at 0.2 M, and stayed stably high at 0.3, 0.4 and 0.5 M. This suggests that adding ions promotes polyphenol adsorption, while the magnitude of added ionic strength has no significant effect on polyphenol adsorption. This may occur because hydrophobic interactions form between the hydrophobic regions of the cellulose polymer and the hydrophobic groups (e.g., aromatic rings) in the polyphenol molecular structure, and these interactions can increase with rising ionic strength [27]. Thus, adding ions can increase the adsorption amount of polyphenols. Phan et al. [28] reached similar conclusions in their study on the adsorption of ferulic acid, catechin, and cyanidin-3-glucoside (Cya-3-glc) onto cellulose under the same range of ionic strengths.

The effect of ionic strength on the particle size of QE-OβG complexes is presented in Table 1. The results show that the average particle size increased with increasing NaCl concentration, indicating enhanced aggregation of the complexes under higher ionic strength conditions. This behavior may be attributed to the influence of salt on the hydration state of the system. The addition of salt alters the binding state of water in the system, enhancing the hydrophobic attraction between QE and OβG molecules, thereby promoting the formation of larger aggregates. However, this interpretation is based on indirect evidence, and further studies are required to elucidate the detailed mechanism.

### 3.3. Structural Characteristics of QE-OβG Complexes

#### 3.3.1. Microstructure

**Figure 2:**
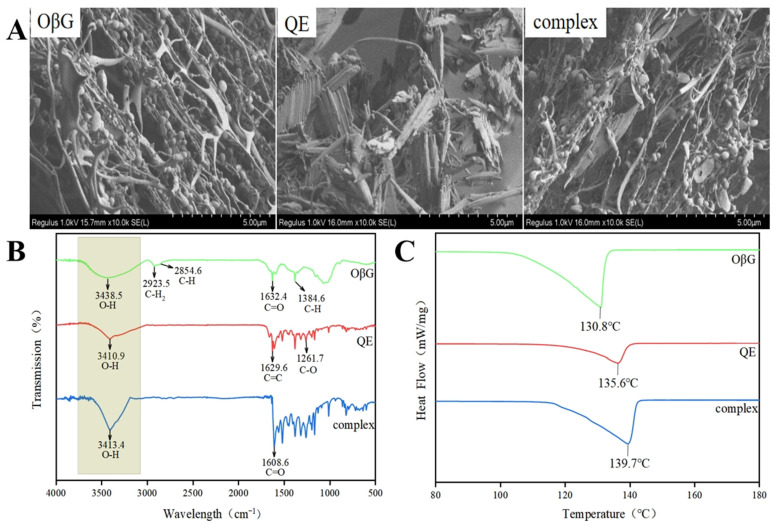
Cryo-SEM images (**A**), FTIR analysis (**B**), and DSC analysis (**C**) of OβG, QE, and QE-OβG complex.

Cold field emission scanning electron microscopy was performed to characterize the microstructure of the composite. Figure 2A shows that when OβG exists alone, it exhibits an interwoven network structure with some particle-like structures distributed within the network; when QE exists alone, it presents a long strip-like or flake-like morphology with needle-like ends. Based on the aforementioned experimental results, when OβG and QE are mixed at a mass ratio of 0.6, the SEM image reveals a morphology where fibrous structures and block-like structures are intertwined and enveloped, collectively forming a complex and interconnected state. This suggests that OβG and QE have aggregated, indicating an interaction between them and the formation of a new composite.

#### 3.3.2. Interaction Forces

The infrared spectra of OβG, QE, and the complex are shown in Figure 2B. The main characteristic absorption peaks of OβG are 2923.5 cm^−1^ and 2854.6 cm^−1^, corresponding to the antisymmetric stretching vibrations of methyl C-H_2_ or methylene C-H [29]; 1632.4 cm^−1^, corresponding to the stretching vibration peak of the carbonyl C=O bond [30]; and 1384.6 cm^−1^, corresponding to the bending vibration peak of C-H [31]. The characteristic absorption peak of QE at 3410.9 cm^−1^ is attributed to the stretching vibration of O-H [32]. The absorption peak at 1629.6 cm^−1^ is associated with the C=C alkene on the benzene ring [33]. The absorption peaks at 1261.7 cm^−1^, 1201.9 cm^−1^ and 1169.6 cm^−1^ can be attributed to the stretching vibration of the C-O bond in the aryl ether ring, the stretching vibration of the C-O bond in the phenol, and the stretching and bending vibrations of the C-O-C bond in the ketone, respectively [34]. The infrared spectrum of the complex essentially retains the characteristic absorption peaks of both components, with peak wavenumbers remaining largely unchanged and peak intensities increasing. The appearance of an absorption peak near 2923.5 cm^−1^ suggests that hydrophobic interactions exist during complexation between QE and OβG [35]. Simultaneously, the peak width at 3413 cm^−1^ for the QE-OβG complex decreased compared to OβG, likely due to hydrogen bonding between the polyphenol hydroxyl group and OβG’s hydroxyl group, reducing the number of free hydroxyl groups [36]. These findings collectively confirm the existence of non-covalent interactions between QE and OβG. The characteristic C=O absorption peak (1632.4 cm^−1^) characteristic of OβG shifts to 1608.6 cm^−1^, indicating that the polar end of OβG may interact with QE. Furthermore, no new infrared absorption peaks appeared for the QE-OβG complex within the scanning range. This suggests that no new covalent bonds formed between QE and OβG. The hydroxyl group of QE likely forms the complex by establishing hydrogen bonds with the polar end of OβG.

#### 3.3.3. Thermal Stability

DSC is a method for determining complex formation in the solid state. The DSC analysis results (Figure 2C) reveal distinct endothermic valleys at 130.8 °C and 135.6 °C for OβG and QE, respectively. These valleys correspond to characteristic transition temperatures for OβG and QE (e.g., depolymerization of molecular chains, melting of crystal structures), directly reflecting their inherent thermal stability. The endothermic peak for the QE-OβG composite appears at 139.7 °C, while the original endothermic peaks of OβG and QE disappear. The composite’s thermal transition temperature is significantly higher than those of OβG and QE. The increased gelatinization temperature of the composite may result from interactions between the hydrophilic hydroxyl groups of OβG and QE, enhancing intermolecular interactions (e.g., hydrogen bonding) and thereby improving thermal stability [9]. Specifically, interactions occur between the hydrophilic hydroxyl groups on OβG and QE molecules. These interactions strengthen intermolecular bonds, requiring the composite to undergo thermal transformation at higher temperatures. From the perspective of thermal behavior, this provides strong evidence that OβG and QE do not form a simple physical mixture but rather a composite with novel thermal properties.

### 3.4. Effects of QE-OβG Complex on the Rheological Properties of Starch

#### 3.4.1. Static Rheological Properties

**Figure 3:**
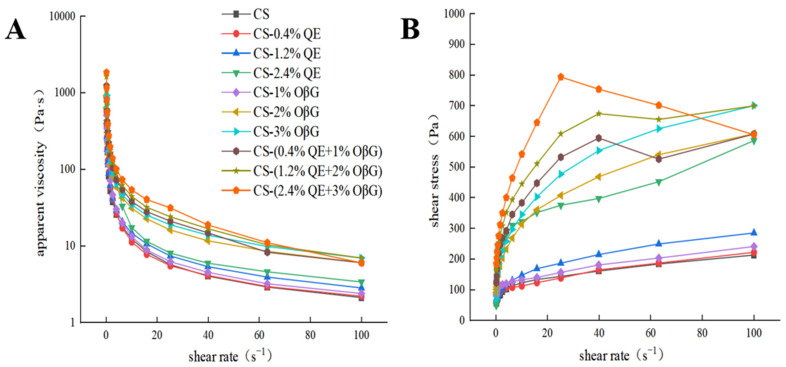
Effect of OβG and QE mass fractions on the static rheological curves of the corn starch system. Note: (**A**) apparent viscosity, (**B**) shear stress.

The effects of OβG and QE mass fractions on the static rheological curves of the corn starch system are shown in Figure 3. As can be seen from Figure 3A, the apparent viscosity decreases significantly with the increase in shear rate; Figure 3B shows that the shear stress increases with the increase in shear rate, and there are significant differences in shear stress among different formulation systems. These results indicate that all corn starch systems exhibit typical shear-thinning behavior [37]. This phenomenon may be attributed to the dissociation of intermolecular forces within the starch system under low shear rates, leading to the orientation and extension of molecular chains; when the shear rate increases to a high level, the entangled structure between molecular chains is completely disrupted, which slows down the decreasing trend of apparent viscosity [38]. The power-law equation was used to fit the system and the R^2^ values of all samples were above 0.9, indicating that this model has high fitting accuracy for the static rheological properties of the corn starch system in this study and can effectively describe its rheological behavior. The flow behavior index n reflects the flow characteristics of the sample: when n = 1, it is a Newtonian fluid; when n < 1, it behaves as a pseudoplastic fluid. In this study, the n values of all samples were much lower than 1, indicating that the systems are pseudoplastic fluids (non-Newtonian fluids). The consistency coefficient K reflects the change in apparent viscosity; a higher K value indicates stronger viscosity of the system. When QE and OβG were added alone, the K value increased significantly with the increase in their mass fractions (Table 2), and the thickening property of the system gradually enhanced, which is consistent with the experimental conclusion of Liu et al. [39]. When OβG and QE were added simultaneously, the K value further increased, indicating an enhancement of the viscoelastic properties of the system. It is speculated that the water-absorbing groups in OβG and QE absorb a large amount of water and form a viscous gel with corn starch, increasing the viscoelasticity of the starch system [40]. Molecules form a denser network structure through hydrogen bonding, molecular entanglement, and other interactions, thereby significantly enhancing the thickening property and pseudoplasticity of the system.

#### 3.4.2. Dynamic Rheological Properties

**Figure 4:**
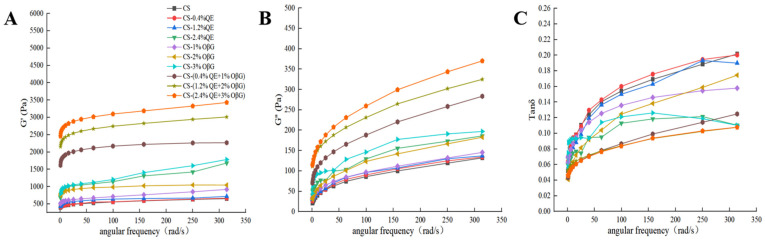
Effect of OβG and QE mass fractions on the dynamic rheological curves of the corn starch system. Note: (**A**) G′, (**B**) G″, (**C**) tanδ.

G′ originates from the change in the conformational entropy of coiled chains and is used to characterize the amount of energy stored during elastic deformation, which is a reversible deformation; meanwhile, G″ reflects the viscous deformation caused by the relative movement of chain segments and molecular chains, representing the energy lost due to viscous deformation, which is an irreversible deformation. The tangent of the loss angle (tanδ) is the ratio of G″ to G′, and is often used to evaluate the viscoelastic behavior of food materials. Generally, tanδ > 1 indicates that the material has good fluid properties, while tanδ < 1 indicates that the material has good solid properties [41]. The dynamic rheological analysis of Figure 4 shows that the dynamic viscoelastic properties of the corn starch system can be systematically characterized by G′, G″, and tanδ. It can be seen that, with an increase in angular frequency, the G′ and G″ values of all systems gradually increase. G′ is always higher than G″, indicating that the system is dominated by elastic behavior and has typical gel characteristics. Specifically, the G′ and G″ values of the system with individually added OβG, the system with individually added QE, and the system with combined addition of OβG and QE are significantly higher than those of pure corn starch. With an increase in the mass fractions of OβG and QE, the increases in G′ and G″ were more obvious, indicating that the addition of OβG and QE effectively enhances the rheological properties of the system.

The tanδ values of all samples are less than 1, which further verifies that the system is dominated by elastic solid properties, consistent with the rheological conclusion that “tanδ < 1 indicates that the elastic behavior of the system is dominant”. At the same time, the tanδ values of different formulation systems fluctuate regularly with angular frequency and addition amount. When QE and OβG were added individually, the tanδ value fluctuated first and then tended to be stable with an increase in OβG content. When OβG and QE were added in combination, the change trend of tanδ reflected the interactive regulatory effect of the two components. This result indicated that the formulation composition could finely regulate the viscoelastic balance of the system. Li et al. [42] pointed out that the inhibitory effects of polysaccharides on starch digestibility are closely related to their own viscosity, which is consistent with the conclusion of this study. The internal mechanism is speculated as follows: macromolecular β-glucan can significantly increase the viscosity of the system, while polyphenols can reduce the contact efficiency between enzymes and gelatinized starch granules. In addition, the diffusion of glucose molecules produced by enzymatic hydrolysis is hindered. These factors work together to ultimately delay the starch digestion process.

### 3.5. Analysis of the Effects of QE-OβG Complex on α-Amylase Activity

#### 3.5.1. Inhibitory Activity

**Figure 5:**
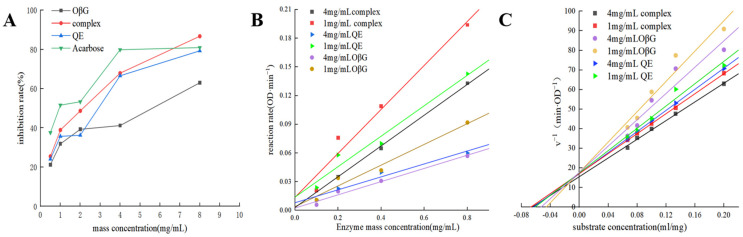
Inhibition rate (**A**), reversibility (**B**), and Lineweaver–Burk plots (**C**) of α-amylase by QE, OβG and QE-OβG complex.

As shown in Figure 5A, in the concentration range of 0~8 mg/mL, the inhibition rates of QE, OβG, and QE-OβG on α-amylase all increase with the increasing mass concentrations, showing an obvious concentration-dependent relationship; among them, the positive control acarbose has the strongest inhibitory activity, achieving a high inhibition rate at low concentrations. The inhibitory ability of QE-OβG is the second strongest, where the increase in inhibition rate with concentration is relatively significant. At a concentration of 8 mg/mL, the inhibition rate of the complex is higher than that of acarbose. The inhibitory activity of QE-OβG is higher than that of QE and OβG, suggesting that combination of the two may produce a certain combined effect. As a single polysaccharide substance, OβG has a weaker inhibitory ability than the flavonoid QE. The half-maximal inhibitory concentrations (IC_50_) of OβG, QE, and the QE-OβG complex on α-amylase are 5.297 mg/mL, 3.32 mg/mL, and 2.629 mg/mL, respectively. The above results indicate that QE, OβG, and QE-OβG all have α-amylase inhibitory activity, among which QE-OβG has the most prominent inhibitory effect, providing an experimental basis for the development of naturally derived starch digestive enzyme inhibitors.

#### 3.5.2. Analysis of the Inhibition Type

The correlation between the enzyme-catalyzed reaction rate of different concentrations of enzyme inhibitors and α-amylase concentration is shown in Figure 5B. It can be seen that the fitting lines of each group approximately pass through the origin of the coordinate system and, with increases in the concentrations of QE, OβG, and the complex, the slope of the corresponding line gradually decreases. This characteristic indicates that the inhibitory effects of QE, OβG, and their complex on α-amylase are all reversible [43]. These substances bind to the enzyme through non-covalent interactions, only reducing the catalytic activity of the enzyme to slow down the reaction rate without causing irreversible inactivation of the enzyme.

The Lineweaver–Burk double reciprocal plots of the reaction rate against substrate concentration in Figure 5C show good linear relationships for all straight lines, which basically intersect at a point in the second quadrant. With increases in the concentrations of QE, OβG, and their complex, the intercept on the abscissa of the straight line decreases, the intercept on the ordinate increases, the corresponding maximum reaction rate Vmax decreases, and the Michaelis constant Km increases. These results indicate that the inhibition types of QE, OβG, and the QE-OβG complex on α-amylase are all mixed-type inhibition. In this inhibition mechanism, the inhibitor can bind to both the free enzyme and the enzyme–substrate complex, thereby simultaneously affecting the binding ability of the substrate to the enzyme and the catalytic efficiency of the enzyme. The changes in Vmax and Km after adding OβG and the complex are greater than those after adding QE, and Km _(OβG)_ > Km _(QE)_ > Km _(QE-OβG)_. The QE-OβG complex exhibited stronger inhibitory activity, which might be due to the presence of QE facilitating the binding of the complex to the enzyme or the enzyme–substrate complex. Meanwhile, the OβG molecule exerted a steric hindrance effect that further prevented the substrate from approaching the active site of the enzyme.

## 4. Conclusions

This study systematically investigated the formation, structural characteristics, and functional properties of QE-OβG complexes, aiming to elucidate their interaction mechanisms and regulatory potential regarding starch digestion. Firstly, the results demonstrated that QE and OβG form stable complexes primarily via non-covalent interactions, including hydrogen bonding and hydrophobic interactions, with the highest binding achieved at a mass ratio of 0.6 under conditions of low pH, 40 °C, and moderate ionic strength (≥0.2 M NaCl). Structurally, the QE-OβG complexes exhibited an intertwined fibrous-block morphology, enhanced thermal stability, and significantly improved viscoelasticity when incorporated into starch systems. Functionally, the QE-OβG complex showed stronger mixed-type reversible inhibition against α-amylase (IC_50_ = 2.629 mg/mL) compared to the individual components. It should be noted that, although the QE-OβG complex showed promising potential in modulating starch digestion-related properties by enhancing system viscosity and inhibiting α-amylase activity, direct evidence supporting its ability to slow starch digestion (e.g., in vitro digestion kinetics, glucose release profiles) is required to confirm its direct effect on starch digestibility in further investigations.

The innovation of this research lies in its systematic extension from the molecular interaction between QE and OβG to the macroscopic functional properties of the complex, which provides a theoretical basis and new perspectives for the design of functional foods based on multi-component interactions. Additionally, these findings offer preliminary insights into the potential applications of QE-OβG complexes in the fields of medicine and food nutrition, especially for the modulation of starch digestion. To promote the practical application of QE-OβG complexes, future studies should focus on validating their functional stability and physiological efficacy in complex food matrices and in vivo digestive models. Furthermore, expanding the research scope to other digestive enzyme systems and exploring the interaction mechanisms of different polyphenol–polysaccharide combinations will facilitate the targeted application of such complexes in the healthy food industry.

## Figures and Tables

**Table 1:** Average particle size, polydispersity index (PDI), and ζ-potential of QE-OβG complex at different mass ratios, pH, temperatures and ionic strengths.

		Average Particle Size (nm)	Polydispersity Index (PDI)	Average Potential (mV)
QE/OβG (*w*/*w*)	0.1	539.67 ± 87.64 d	0.36 ± 0.1 d	−10.83 ± 0.15 a
	0.2	810.33 ± 53.8 c	0.55 ± 0.11 cd	−15.73 ± 0.71 c
	0.4	1007.33 ± 322.01 bc	0.61 ± 0.04 bc	−14.37 ± 0.4 bc
	0.6	1693.67 ± 101.28 b	0.79 ± 0.19 ab	−14.37 ± 0.5 bc
	0.8	2033.33 ± 91.57 a	0.89 ± 0.18 a	−13.53 ± 0.84 b
	1	2345 ± 263.74 a	1 ± 0 a	−17.77 ± 1.46 d
pH	7	773.33 ± 90.42 c	0.82 ± 0.04 a	−9.98 ± 0.66 a
	6	877 ± 3.61 c	0.75 ± 0.22 a	−5.15 ± 0.35 b
	5	948.33 ± 79.01 bc	0.59 ± 0.16 a	−3.19 ± 0.34 c
	4	1032.67 ± 143.98 bc	0.59 ± 0.15 a	−2.63 ± 0.05 c
	3	1575 ± 137.48 b	0.42 ± 0.31 a	−1.35 ± 0.21 d
	2	2049 ± 76.97 a	0.46 ± 0.37 a	1.42 ± 0.14 e
Temperature (°C)	4	534.04 ± 157.45 b	0.76 ± 0.03 b	−17.77 ± 0.76 bc
	25	843.55 ± 104.93 b	0.83 ± 0.07 b	−19.3 ± 1.35 c
	40	785.68 ± 536.19 b	0.82 ± 0.15 b	−18.03 ± 1.17 bc
	55	723.67 ± 358.53 b	0.84 ± 0.02 b	−16.97 ± 0.72 b
	70	753.28 ± 182 b	0.85 ± 0.07 b	−17.13 ± 0.51 b
	85	433.33 ± 212.44 a	1 ± 0 a	−12.23 ± 0.84 a
Ionic strength (M)	0	866 ± 667.3 a	0.82 ± 0.16 a	−15.60 ± 0.95 c
	0.1	613.33 ± 914.16 a	0.95 ± 0.09 a	−2.99 ± 0.74 ab
	0.2	899.33 ± 100.76 a	0.85 ± 0.09 a	−4.78 ± 2.30 b
	0.3	902.67 ± 189.43 a	0.79 ± 0.10 a	−4.10 ± 1.89 ab
	0.4	956 ± 672.58 a	0.80 ± 0.11 a	−2.43 ± 0.86 a
	0.5	1020.33 ± 170.57 a	0.73 ± 0.11 a	−2.01 ± 0.60 a

Notes: Different letters in a single column denote significant differences at *p* < 0.05.

**Table 2:** Effect of OβG and QE mass fractions on the power-law equation fitting parameters for the static rheology of corn starch systems.

Sample	K	n	R^2^
CS	80.51 ± 9.34 ^d^	0.1892 ± 0.0020 ^f^	0.9907
CS-0.4% QE	85.27 ± 14.95 ^d^	0.1762 ± 0.0013 ^g^	0.9664
CS1.2% QE	97.31 ± 8.21 ^d^	0.2038 ± 0.0008 ^e^	0.9164
CS-2.4% QE	183.90 ± 16.37 ^bc^	0.2600 ± 0.0013 ^b^	0.9157
CS-1% OβG	108.89 ± 11.99 ^d^	0.1345 ± 0.0161 ^h^	0.9924
CS-2% OβG	163.90 ± 14.20 ^c^	0.2821 ± 0.0010 ^a^	0.9961
CS-3% OβG	187.47 ± 43.93 ^bc^	0.2808 ± 0.0080 ^a^	0.9905
CS-(0.4% QE + 1% OβG)	217.65 ± 19.48 ^b^	0.2497 ± 0.0100 ^c^	0.9861
CS-(1.2% QE + 2% OβG)	259.76 ± 39.05 ^a^	0.2326 ± 0.0105 ^d^	0.9895
CS-(2.4% QE + 3% OβG)	290.87 ± 30.00 ^a^	0.2321 ± 0.0026 ^d^	0.9396

Notes: Different letters in a single column denote significant differences at *p* < 0.05.

## Data Availability

The original contributions presented in this study are included in the article/Supplementary Materials. Further inquiries can be directed to the corresponding author.

## Supplementary Materials

The following supporting information can be downloaded at: https://www.mdpi.com/article/10.3390/foods15101825/s1, Figure S1: preparation process of the QE-OβG complex.

## Abbreviations

The following abbreviations are used in this manuscript:

QE	quercetin
OβG	oat β-glucan
DNS	3,5-dinitrosalicylic acid
PDI	polydispersity index
Cryo-SEM	cryo-scanning electron microscopy
FTIR	Fourier transform infrared spectroscopy
DSC	differential scanning calorimetry
G′	storage modulus
G″	loss modulus
tanδ	loss tangent
IC_50_	half-maximal inhibitory concentration
