# Supplementary figures and images for: Formation, Structural Characteristics and Functional Properties of Quercetin–Oat β-Glucan Complex
Source: Foods. 2026 May 21;15(10):1825. doi: 10.3390/foods15101825 (PMC13205155; doi:10.3390/foods15101825)

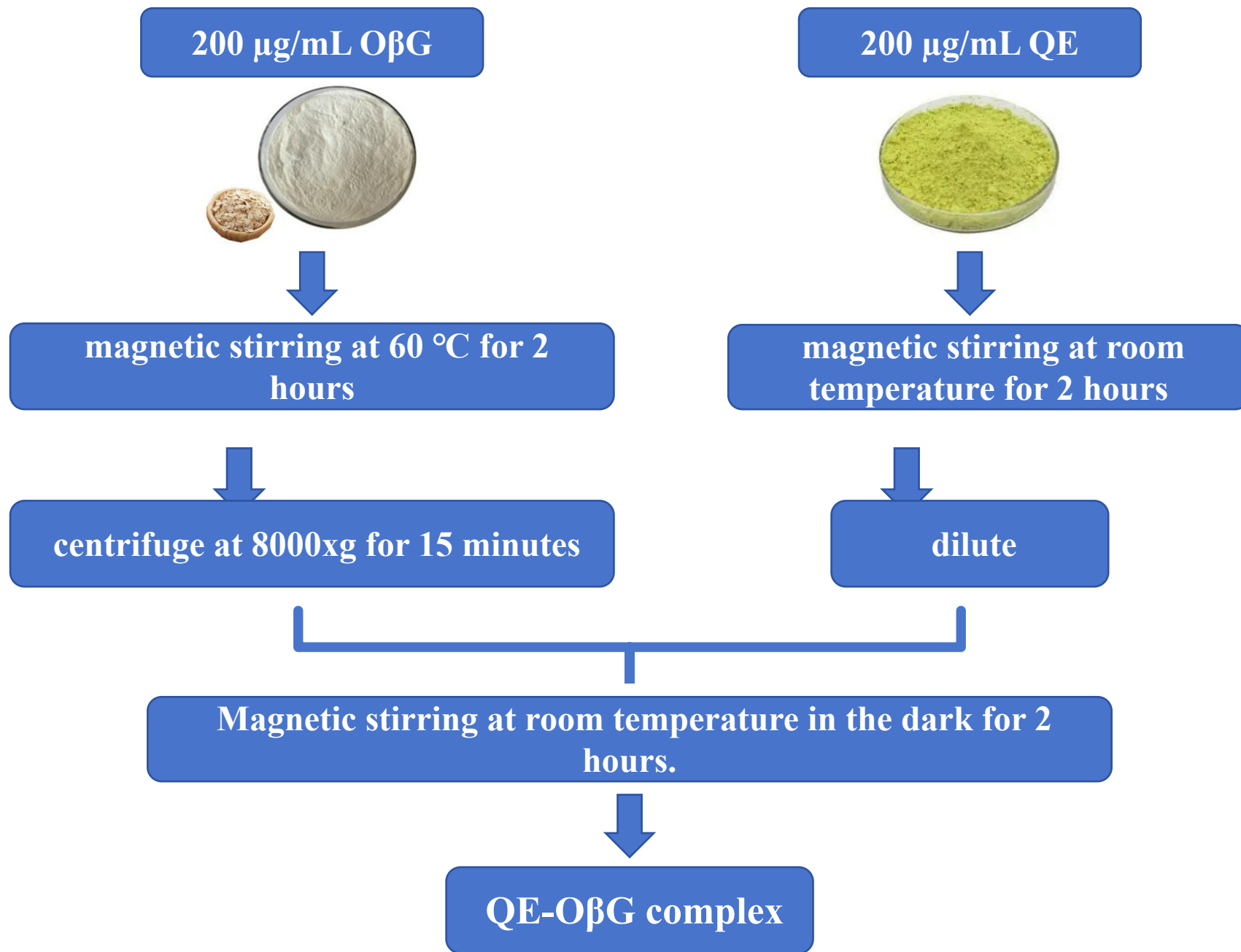

**Figure S1.** preparation process of the QE-O $\beta$ G complex

Supplement: Supplementary file 1 [file foods-15-01825-s001.zip › foods-4221648-supplementary.pdf]
